# Chromatin remodeling agent trichostatin A: a key-factor in the hepatic differentiation of human mesenchymal stem cells derived of adult bone marrow

**DOI:** 10.1186/1471-213X-7-24

**Published:** 2007-04-02

**Authors:** Sarah Snykers, Tamara Vanhaecke, Ann De Becker, Peggy Papeleu, Mathieu Vinken, Ivan Van Riet, Vera Rogiers

**Affiliations:** 1 Dept. Toxicology., Vrije Universiteit Brussel, Laarbeeklaan 103, B-1090, Brussels, Belgium; 2 Dept. Medical Oncology and Hematology, Stem Cell Laboratory, Academic. Hospital, Vrije Universiteit Brussel, Laarbeeklaan 101, B-1090, Brussels, Belgium

## Abstract

**Background:**

The capability of human mesenchymal stem cells (hMSC) derived of adult bone marrow to undergo *in vitro *hepatic differentiation was investigated.

**Results:**

Exposure of hMSC to a cocktail of hepatogenic factors [(fibroblast growth factor-4 (FGF-4), hepatocyte growth factor (HGF), insulin-transferrin-sodium-selenite (ITS) and dexamethasone)] failed to induce hepatic differentiation. Sequential exposure to these factors (FGF-4, followed by HGF, followed by HGF+ITS+dexamethasone), however, resembling the order of secretion during liver embryogenesis, induced both glycogen-storage and cytokeratin (CK)18 expression. Additional exposure of the cells to trichostatin A (TSA) considerably improved endodermal differentiation, as evidenced by acquisition of an epithelial morphology, chronological expression of hepatic proteins, including hepatocyte-nuclear factor (HNF)-3β, alpha-fetoprotein (AFP), CK18, albumin (ALB), HNF1α, multidrug resistance-associated protein (MRP)2 and CCAAT-enhancer binding protein (C/EBP)α, and functional maturation, i.e. upregulated ALB secretion, urea production and inducible cytochrome P450 (CYP)-dependent activity.

**Conclusion:**

hMSC are able to undergo mesenchymal-to-epithelial transition. TSA is hereby essential to promote differentiation of hMSC towards functional hepatocyte-like cells.

## Background

The ever-increasing number of new chemical entities (NCEs) has prompted the development of research methods for rapid screening. Present research efforts are therefore directed at developing a series of *in vitro *assays that could be applied for preclinical screening of NCEs. By utilizing *in vitro *models, pharmaceutical companies attempt to reduce clinical failure rates by accurately evaluating efficacy and safety much earlier in the drug discovery process [1]. As the liver is the principal organ for xenobiotic biotransformation and thus a key target for drug-induced toxicity, *in vitro *models to predict drug metabolism are preferably derived from human liver tissues. However, the use of human hepatocytes is limited by scarcity of intact and fresh human liver tissue [2,3]. In addition, primary hepatocyte cultures still cope with a progressive occurrence of dedifferentiation, resulting in a relative short life-span and a rapid decline of liver-specific functions [4-7]. Different alternatives have been explored to overcome these difficulties, including the use of human stem cells. *In vivo *as well as *in vitro *studies have provided evidence that stem cells can overcome germ lineage restrictions and express molecular characteristics of cells of different tissue origin [8,9].

The best characterized stem cell compartment is the bone marrow consisting of two stem cell populations, referred to as the hematopoietic and mesenchymal stem cells [9]. The latter was first described by Friedenstein *et al*. as a population of cells isolated from the bone marrow and capable of differentiation into bone, adipocytes, chondrocytes, osteoblasts, osteoprogenitors, skeletal myocytes, tendon and bone marrow stromal cells [10,11].

Schwartz *et al*. described a novel population of cells within postnatal rat bone marrow, named multipotent adult progenitor cells (MAPC), that were not only capable to differentiate into most mesodermal cell types, but also into functional neuroectodermal and endodermal (hepatocytes) cell types [12].

In the present study, we investigated whether hMSC also have the potential to differentiate *in vitro *into functional hepatocytes. Therefore, hMSC were cultivated in the presence of 'hepatogenic' factors, added either as a cocktail (FGF-4, HGF, ITS and dexamethasone) or, more innovative, in a sequential manner closely reflecting their temporal expression during *in vivo *hepatogenesis (FGF-4, followed by HGF, followed by a combination of HGF, ITS and dexamethasone). Next, giving the promising differentiation-inducing properties of histone deaceylase inhibitors (HDAC-I) in primary cultures of adult hepatocytes [13-16] and hepatoma cell lines [17-19], we additionally examined the effect of TSA, a potent and specific hydroxamic acid-based HDAC-I, on hMSC cultures.

## Results

### Characterization of culture-expanded hMSC

hMSC were cultivated from the mononuclear cell fraction of bone marrow samples obtained from healthy donors. To ensure removal of contaminating hematopoietic cells, cells were selected, based on plastic adherence, and passaged 4 times prior to further use.

**Figure 1:**
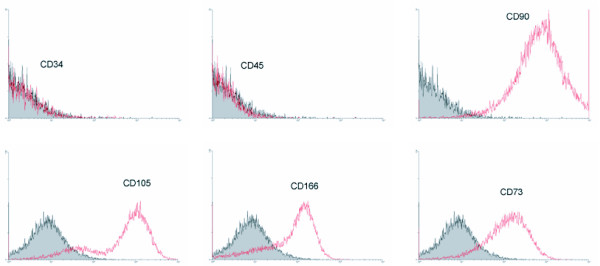
*Immunophenotypic analysis of culture-expanded hMSC*. Flow cytometry analysis was performed for CD34, CD45, CD90, CD105, CD166 and CD73. The results shown are representative for 5 independent experiments.

**Figure 2:**
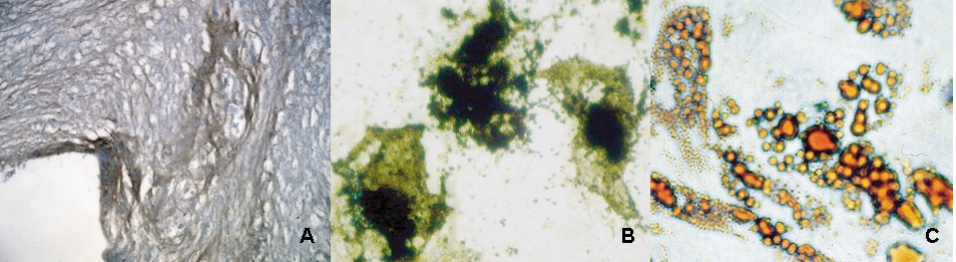
*Mesodermal differentiation potency of culture-expanded hMSC*. Characterization of culture-expanded hMSC upon exposure to specific (A) chondrogenic, (B) osteogenic and (C) adipogenic agents, respectively. Chondrogenic, adipogenic and osteogenic differentiation potential were confirmed by means of (A) immunohistochemical staining of collagen type II fibers, (B) Von Kossa and (C) Oil Red O staining, respectively. 64 × 10 original magnification. Stainings shown are representative for at least 5 separate experiments.

To ascertain that the culture-expanded cells were genuine MSC, both their phenotype and mesodermal differentiation potential upon exposure to mesenchymal-supportive conditions, i.e. chondrogenic, osteogenic and adipogenic-specific agents, were examined (Fig. 1). Immunophenotypic analysis revealed expression of CD73, CD90, CD105 and CD166 and no expression of CD34 and CD45 (Fig. 1). Von Kossa and Oil-Red O staining confirmed the presence of calcium deposits, characteristic for osteogenic cells, and lipid droplets, respectively. Immunohistological staining for collagen type II verified chondrogenic differentiation (Fig. 2). In summary, these results indicate that the expanded cells have the basic properties of genuine MSC.

### Differentiation of hMSC under 'hepatogenic-supporting' conditions

To evaluate whether hMSC could differentiate into endodermal cells, as described earlier for MAPC [12], cells were exposed to hepatogenic agents, either as a cocktail [12] or in a sequential manner [20], and analyzed by means of microscopic analysis and specific cytological stainings.

#### Optimization of culture conditions

A prerequisite for differentiation *in vitro *is cell cycle arrest. In preliminary experiments, we found that, in the presence of cytokines and growth factors, a non-proliferative status is only obtained at 100% confluence. Therefore, the latter cell density was further applied in all differentiation experiments.

#### Endogenic differentiation upon sequential exposure of hMSC to hepatogenic factors

**Figure 3:**
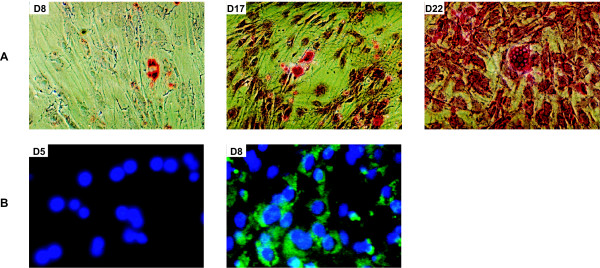
*Differentiation potential of hMSC upon sequential exposure to hepatogenic agents*. Upregulated (A) glycogen storage and (B) CK18 expression was shown by means of PAS-staining and immunofluorescence, respectively. A: 10 × 10 original magnification; B: 32 × 10 original magnification. Stainings shown are representative for at least 5 separate experiments.

In contrast to MAPC [12], hMSC did not gain typical morphologic and phenotypic characteristics of hepatocytes upon exposure to a cocktail of hepatogenic factors. On the opposite, sequential treatment of hMSC with FGF-4, HGF, ITS and dexamethasone in a time-dependent order closely reflecting their secretion pattern during *in vivo *liver ontogeny ('sequential condition') resulted in upregulation of glycogen uptake from day 8 onwards (Fig. 3A). From days 8–9 onwards, cells also expressed CK18 (Fig. 3B), a cytoskeletal filament present in hepatocytes. The morphology of the differentiated cells, however, did not resemble hepatocytes.

### TSA, a trigger for functional maturation of endogenic differentiating hMSC

In an attempt to enhance endogenic differentiation of hMSC, TSA, a selective and reversible HDAC-I [13-19], was added to the culture media in a concentration of 1 μM after exposure of the cells to the hepatogenic factors.

#### Optimization of culture conditions

Besides the state of growth arrest, 'time' is often critical for successful 'trans'differentiation of (progenitor/stem) cells in epigenetic regulating processes [21]. More specifically, promotion of 'trans'differentiation into a specific lineage by means of HDAC inhibition often requires selective pre-stimulation of the cells. In preliminary experiments, we searched for the most optimal differentiation conditions. Hereto, we examined the effect of the hepatic inducing effects of (1) FGF-4, HGF, ITS and dexamethasone, added alone and in different combinations (e.g. FGF-4, HGF/HGF, ITS/HGF, dexamethasone/ITS, dexamethasone/ITS, HGF etc.), and (2) pre- and post-treated with the HDAC inhibitor TSA. Out of the results obtained, we found that 1 μM TSA, when added exclusively to MSC at 100% confluence and without pretreatment with FGF-4 and HGF, was not effective in stimulating mesenchymal-to-hepatic transition. The same applies when these cells were treated with FGF-4 and HGF afterwards (data not shown). In addition, treatment of undifferentiated hMSC with 1 μM TSA resulted in massive cell death and cell detachment (data not shown). Suppression of proliferation, in parallel with cell survival and long-term cultivation, were only obtained upon pre-stimulation of the cells with hepatogenic factors for at least 6 days prior to addition of 1 μM TSA (see Additional files 1, 2, 3). In spite of the good long-term cell survival, about 20% of the TSA-treated cells underwent apoptosis (see Additional file 3). In the present study, the most appropriate conditions for transdifferentiation comprised exposure of hMSC to **1 μM TSA, starting on the 6^th ^day of exposure to hepatogenic cytokines and growth factors; these treatments are further referred to as 'cocktail TSA' and 'sequential TSA' conditions**.

#### Morphological features

**Figure 4:**
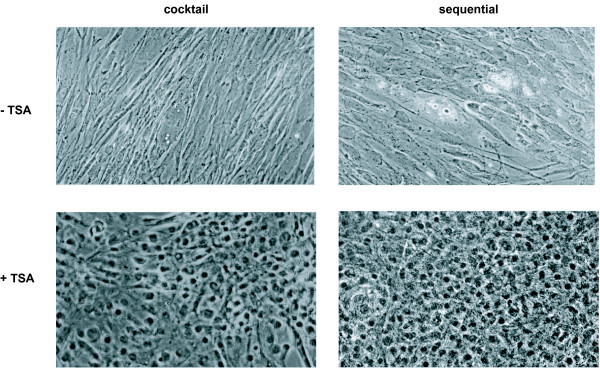
*Cell morphology*. Light-microscopic analysis of 17-day old sequentially (+/-1 μM TSA) and cocktail (+/-1 μM TSA)-exposed hMSC; 20 × 10 original magnification, phase contrast.

Interestingly, upon exposure to 1 μM TSA, added from day 6 on to differentiating hMSC, cells in about 25% of all bone marrow samples tested underwent a drastic morphological change, regardless of the culture condition used (Fig. 4). After 3–5 days, both sequential TSA- and cocktail TSA-treated hMSC formed epithelioid cells with a clear round nucleus. Fibroblastic cells, however, persisted throughout the culture time, particularly upon exposure to the cocktail TSA-condition (Fig. 4), whereas a more homogeneous cell population was obtained in the sequential TSA set-up.

#### Liver-specific protein expression

**Figure 5:**
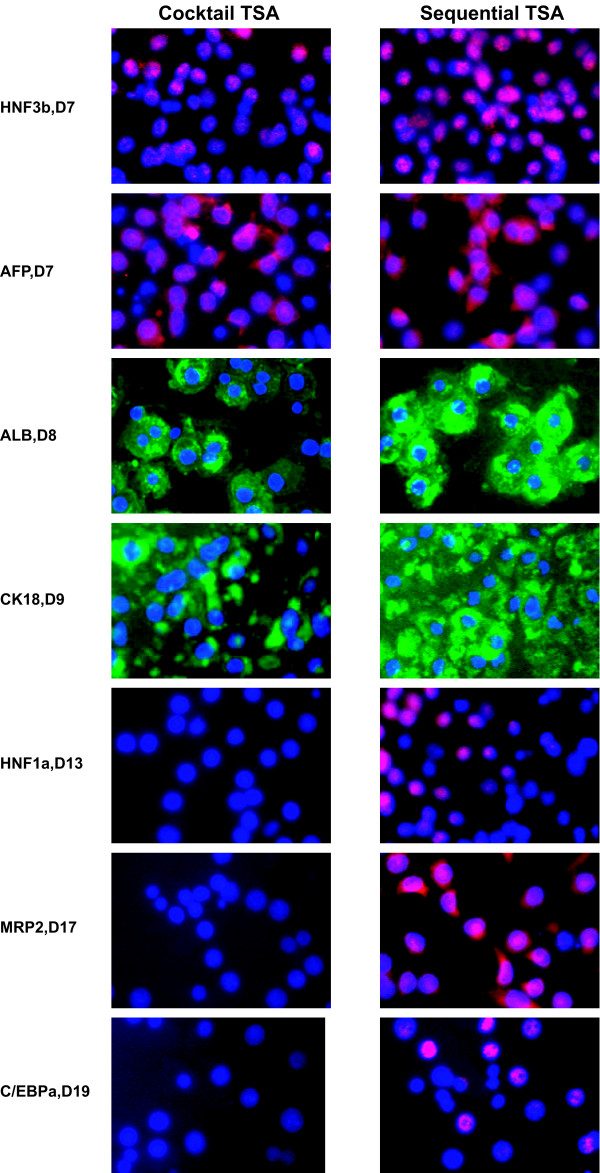
*Characterization of hepatic differentiation of sequentially TSA- and cocktail TSA-exposed hMSC*. Immunofluorescence was performed for HNF3β-cy3, AFP-FITC, ALB-FITC, CK18-FITC, HNF1α-cy3, MRP2-cy3 and C/EBPα-cy3; 32 × 10 original magnification. Stainings shown are representative for at least 4 separate experiments.

To evaluate whether these morphological changes were associated with enhanced differentiation towards hepatocyte-like cells, analysis of early (HNF3β, AFP), mid-late (ALB, CK18) and late (HNF1α, MRP2 and C/EBPα) hepatic markers was performed at the protein level. In fact, sequentially TSA-exposed cells progressively reached an adult expression pattern: next to CK18 (57 ± 8% on day 9), cells also stained positive for HNF3β (76 ± 2% on day 7), AFP (77 ± 4% on day 7), ALB (87 ± 2% on day 8), HNF1α (79 ± 1% on day 13), MRP2 (78 ± 1% on day 17) and C/EBPα (50 ± 3% on day 19) and this in a specific chronological order (Fig. 5). In line with the results at the morphological level, cocktail TSA-treatment supported hepatic differentiation only to a limited extent. More specifically, throughout the whole culture time, cells failed to express HNF1α and C/EBPα, both late liver-enriched transcription factors. Also, the major hepatic drug transporter MRP2 was not expressed (Fig. 5).

#### Hepatic functionality

To evaluate whether these hepatocyte-like cells derived from adult human bone marrow also acquired typical functional hepatic features, ALB secretion, ammonia metabolism and inducible CYP-dependent activity were next analysed.

##### ALB secretion

**Figure 6:**
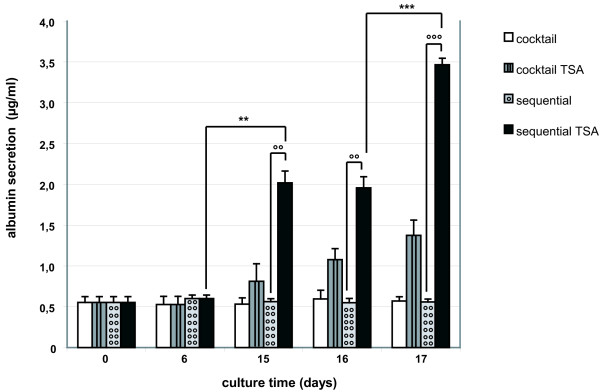
*ALB secretion into the medium was measured by ELISA*. hMSC were differentiated in sequential (+/-1 μM TSA) and cocktail (+/-1 μM TSA) conditions. The results shown are representative for 5 independent experiments. **, ***: ALB-secretion of hMSC cultivated in the sequential TSA-condition is significantly upregulated during culture time, p < 0.01 and p < 0.001 (Student's t-test). °°, °°°: ALB-secretion of hMSC cultivated in the sequential TSA-condition is significantly higher than in the sequential set-up, p < 0.01 and p < 0.001 (Student's t-test).

1 μM TSA, added to sequentially-treated hMSC from day 6 onwards (sequential TSA-condition), significantly upregulated the ALB secretion rate from day 15 onwards (p < 0.01, Oneway Anova and Student's t-test) when compared with regular sequential cultures (Fig. 6). A limited upregulation of the ALB secretion was also detected upon cocktail TSA-exposure. The raise, however, was not significant and less distinctive than observed after sequential TSA-treatment. hMSC cultivated without TSA did not secrete ALB (Fig. 6).

##### Ureogenesis

**Figure 7:**
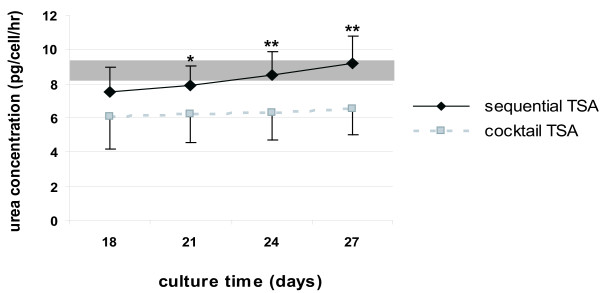
*Urea production in sequentially TSA- and cocktail TSA-exposed hMSC*. Gray area represents urea levels, produced by 4 hours-cultured adult hepatocytes. The graph is representative for 4 separate experiments, each performed in duplicate. **: Urea production significantly differs among sequentially TSA- and cocktail TSA-exposed hMSC with p < 0.01 (Student's t-test).

Upon sequential TSA-exposure, the urea production increased in a time-dependent manner, reaching similar levels as measured in 4 hours-cultured adult hepatocytes after 24–27 days differentiation (Fig. 7). Cocktail TSA-exposed hMSC could also synthesize urea; however levels remained stable and were, even at peak production, significantly lower (i.e. 29%) (p < 0.01; Student t-test) than the sequential TSA condition (Fig. 7). In contrast, sequentially and cocktail-exposed cells, cultivated without TSA, did not metabolize ammonia (data not shown).

##### CYP-dependent activity and inducibility

**Figure 8:**
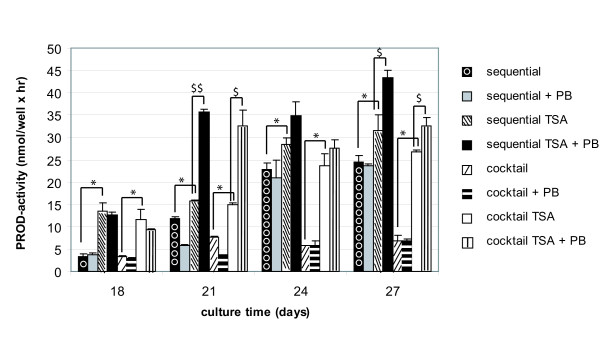
*PROD-activities and responsiveness to 1 mM PB in sequentially (+/-1 μM TSA) or cocktail (+/-1 μM TSA)-exposed hMSC*. PB was added daily, starting on day 18. The graph is representative for 5 separate experiments, each, performed in duplicate. PB, phenobarbital. *: PROD-activity of sequentially TSA- and cocktail TSA-exposed hMSC is significantly higher than of sequentially- and cocktail-exposed hMSC with p < 0.05 (Student's t-test). $,$$: PB significantly induced PROD-activity of sequentially TSA- and cocktail TSA-exposed hMSC with p < 0.05 and p < 0.01, respectively (Student's t-test).

**Figure 9:**
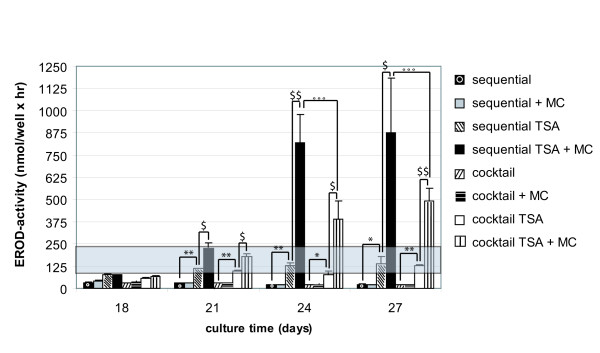
*EROD-activities and responsiveness to 2 μM MC in sequentially (+/-1 μM TSA) or cocktail (+/-1 μM TSA)-exposed hMSC*. MC was added daily, starting on day 18. Gray area represents EROD-activity measured in 4 hours-cultured adult hepatocytes, not treated with TSA. The graph is representative for 5 separate experiments, each performed in duplicate. MC, 3-methylcholantrene. *, **: EROD-activity of sequentially TSA- and cocktail TSA-exposed hMSC is significantly higher than of sequentially- and cocktail-exposed hMSC with p < 0.05 and p < 0.01, respectively (Student's t-test). $,$$: MC significantly induced EROD-activity of sequentially TSA- and cocktail TSA-exposed hMSC with p < 0.05 and p < 0.01, respectively(Student's t-test). °°°: CYP1A1/2-inducibility significantly differs among sequentially TSA- and cocktail TSA-exposed hMSC with p < 0.001 (Student's t-test).

Sequentially-exposed hMSC expressed functionally active CYP2B6 (Fig. 8). In contrast, no CYP-dependent activity could be measured upon cocktail-treatment. Additional exposure to 1 μM TSA significantly promoted the metabolising capacity of CYP2B6 and CYP1A1/2 from days 18 and 21 onwards, respectively, regardless of the experimental set-up (p < 0.05; Student's t-test) (Figs. 8, 9). By day 21, ethoxyresorufin-O-deethylase (EROD)-activity even increased towards levels measured in 4 hours-cultivated primary hepatocytes (Fig. 9). Comparable to the results obtained for ALB and urea secretion, throughout the culture time, the metabolising capacity of sequential TSA-treated cells exceeded that of cocktail TSA-exposed cells (Figs. 6, 7, 8, 9).

The potential to induce CYP-dependent monooxygenases is considered as one of the most representative functional parameters to evaluate the adult hepatic phenotype [22,23]. Therefore, the responsiveness of both CYP1A1/2 and CYP2B6 to their respective prototype inducers 3-methylcholantrene (MC) and phenobarbital (PB) was studied in parallel. Interestingly, only TSA-treated cells exhibited inducible CYP-activities (Figs. 8, 9). More specifically, upon TSA-treatment pentoxyresorufin-O-dealkylase (PROD)-activities were significantly induced up to 2.2 fold after 3 days exposure to PB (i.e. on day 21), regardless of the experimental configuration (p < 0.05; Student's t-test). The inducibility persisted throughout the culture time (Fig. 8). In addition, a significant 6.4 fold CYP1A1/2-dependent response to MC was observed on days 24–27 in the sequential TSA-model (p < 0.05; Student's t-test). Cocktail TSA-treated cells also showed inducible EROD-activity, but to a significantly lesser extent (p < 0.001, Student's t test) (Fig. 9).

An overview of all experiments performed along with their outcome per condition is summarized in table 1.

**Table 1:** Summary of all characterization experiments performed along with their outcome.

	**SEQUENTIAL**	**COCKTAIL**	**SEQUENTIAL TSA**	**COCKTAIL TSA**
**ENDOGENIC DIFFERENTIATION**				
**Cytological staining**				
PAS	++	-	NT	NT
**Immunofluorescence**				
AFP, HNF3β, ALB, CK18, HNF1α, C/EBPα, MRP2	-	-	+++	++
**Functional analysis**				
ALB secretion	-	-	+++	++
Ureogenesis	-	-	+++	++
EROD activity	-	-	+++	++
Inducible EROD activity	-	-	+++	++
PROD activity	+	-	+++	++
Inducible PROD activity	-	-	+++	++

(-): negative; (+),(++),(+++): weak positive, positive, strong positive, respectively; NT: not tested.

### TSA causes accumulation of acetylated histone H4

The question, however, still remains whether the improved hepatic differentiation of hMSC, upon exposure to 1 μM TSA, is mediated by TSA-induced HDAC inhibition. Since histone H4 is a known target of TSA (21), the acetylated status of this protein was analysed by means of immunofluorescence.

**Figure 10:**
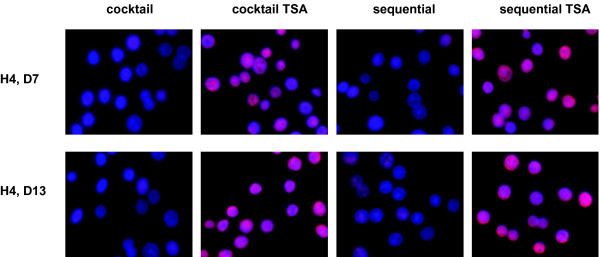
*TSA-induced HDAC inhibition*. Acetylated status of histone H4 in sequentially (+/-1 μM TSA) and cocktail (+/-1 μM TSA)-treated hMSC; 32 × 10 original magnification. *Differentiation potential of bone-marrow-derived hMSC under sequential-, sequential TSA-, cocktail- and cocktail TSA-conditions*.

Histone H4 was indeed clearly hyperacetylated in both the cocktail TSA and sequential TSA set-up when compared to the appropriate controls(Fig. 10). Affecting the acetylated status of histone proteins could thus be proposed here to regulate cell fate.

## Discussion

Hepatocyte-based *in vitro *models are often-used tools for pharmaco-toxicological research and could also become an interesting tool for regulatory testing of xenobiotics [1]. A serious drawback, however, is the occurrence of cellular dedifferentiation [5-7] and the shortage of cells of human origin. The use of stem cells could offer a solution. It is well recognized that in adult mammals, including humans, a number of tissues are continuously regenerated from immature cells (i.e. skin, intestinal epithelia, blood, the olfactory bulb in the brain) [8-12,24,25]. The interest in adult stem cells has in particular been triggered by the numerous ethical dilemmas surrounding the use of embryonic stem cells in preclinical and clinical research [26]. Consequently, successful isolation, cultivation and manipulation of multipotent stem cells from non-embryonic origin could provide researchers with an unlimited human cell source for cell and organ development studies, preclinical pharmaco-toxicological research and regulatory testing.

In the current study, we investigated the *in vitro *endodermal potency of hMSC. hMSC were hereto exposed to well-defined hepatogenic agents, added either as a cocktail ('cocktail condition') or, more innovative, in a sequential time-dependent order as seen during liver embryogenesis ('sequential condition').

Exposure of hMSC to a cocktail of hepatogenic factors failed to induce mesenchymal-to-epithelial transition. In contrast, sequential treatment with these hepatogenic agents ('sequential condition') not only induced expression of the mid-late hepatic marker CK18 from day 8 onwards but also resulted in hepatic functionality, as evidenced by upregulated glycogen storage from days 8 to 22 and acquisition of CYP2B6-activity, though not inducible, from day 21 onwards. However, cells did not morphologically resemble adult hepatocytes.

As a trigger for further differentiation of hMSC towards the endodermal lineage, TSA, known as a proliferation-inhibiting and differentiation-inducing agent in primary hepatocytes and hepatoma cell lines [14-19], was introduced into the present culture media. Previously, our group showed that exposure of 1 μM TSA to primary cultured hepatocytes induced cell cycle arrest during G0/G1 and G1/S phase in EGF-stimulated cells, significantly retarded apoptosis and improved cell junction intercellular communication and xenobiotic biotransformation [13-16,27,28]. These findings encouraged us to expose differentiating hMSC to TSA as well. In fact, upon addition of TSA, hMSC of 1/4 of the bone marrow samples tested, underwent a mesenchymal-to-hepatic transition. More specifically, differentiating TSA-exposed cells adopted an epithelial morphology, expressed liver-specific proteins and acquired functional maturation, regardless of the experimental set-up, although to a different degree. A rather homogeneous population of epithelioid cells was obtained upon sequential TSA-treatment. About 70% of these differentiated cells expressed early (HNF3β, AFP), mid-late (ALB, CK18) and late (HNF1α, MRP2 and C/EBPα) hepatocyte markers in a ranking order comparable to their expression pattern during *in vivo *hepatogenesis. Indeed, AFP expression is first detected in embryonic endoderm around E8.5 [29] and precedes ALB, HNF1α and MRP2 expression, detected around E9.5, E10.5 and E16, respectively [30-32].

Furthermore, hepatic metabolic functions, including ALB secretion, urea production, and inducible CYP-activity, were manifested exclusively in TSA-treated cells and most prominently under the sequential TSA-conditions. More specifically, TSA significantly upregulated ALB secretion, urea synthesis and CYP1A1/2-activities to levels comparable to those found in 4 hours- to 2 days-old monolayer cultures of adult hepatocytes [15]. In line with the behaviour of adult hepatocytes, both *in vitro *and *in vivo*, responses to the prototype inducers MC and PB were pronounced and discrete, respectively; however, levels of induction, measured in the bone marrow-derived hepatocyte-like cells remained lower than in true adult hepatocytes.

On the other hand, cocktail TSA-treated cells also featured hepatic phenotype and function, but less differentiated, which corresponds well to the persistence of processes throughout the culture time.

Recently, the hepatic differentiation potential of MSC, derived from bone marrow, adipose tissue and umbilical cord blood, has been affirmed by showing expression of distinct hepatocyte markers and functions, including ALB and urea secretion, glycogen storage and LDL uptake, upon *in vitro *conditioning (i.e. exposure to hepatogenic agents) of the cells [33-41]. In contrast to our findings, a functional hepatic phenotype was generally not seen before 3.5–4 weeks post-stimulation. In addition, no inducible CYP-dependent **activity **was reported, which is considered to be a key determinant of the functional hepatic phenotype, and a prerequisite for applicability of the cells as *in vitro *models for preclinical drug testing. Only one study described inducible Cyp2B6 expression at both the mRNA and protein-level [34] but not before 4 to 6 weeks preconditioning of the cells [34]. In contrast, our TSA-exposed cells already possessed PROD (CYP2B6)-dependent **activity **and -inducibility upon 21 days differentiation.

In line with our results, Seo et al., 2005 showed enhanced hepatic differentiation upon addition of 0.1% dimethylsulfoxide (DMSO) to human adipose tissue-derived stromal cells (hADSC), prestimulated for 10 days with a cocktail of hepatogenic cytokines [33]. The mechanism underlying the hepatic (trans)differentiation-inducing effects of DMSO have not yet been fully elucidated. However, like TSA, histone hyperacetylation-inducing effects have been ascribed to DMSO as well [42].

Other research groups showed hepatic differentation of hMSC upon stimulation to hepatogenic cytokines exclusively, added either as a mixture [FGF-4+HGF [35,36], separately [HGF [40]; HGF followed by oncostatin M (OSM) [34,37] or as a combination thereof [FGF+HGF followed by OSM [41]]. Conform to our experience, the results shown by Lange et al., 2005 disagreed with those reports [38]. The reasons underlying these controversial results are unknown. However, part might be ascribed to subtle differences in hMSC isolation/expansion techniques, and microenvironment (growth factors, cytokines, medium additives, cell-cell/cell-matrix contact, etc.), playing a decisive role in the specific and directed differentiation of stem/progenitor cells. Additionally, it should be taken into consideration that the plasticity of stem/progenitor cells highly depends on the tissue harvest site and the donor's profile (age, gender, body mass index, lifestyle, drug intake, etc.). The differentiating potential of bone marrow-derived MSC, in fact, decreases with age [43]. In our study, most bone marrow samples were harvested from elderly patients, which might also form a part of the answer. In addition, the hepatic differentiation efficiency of all bone marrow samples tested in the present study yielded 25%, postulating influence of donor-related variability as well. The elucidation of all factors involved seems thus of primary importance and will be explored in future experiments.

## Conclusion

The developmental process of hepatocytes towards a fully polarized and functional phenotype is known to depend on a complex network of transcriptional and posttranscriptional regulatory events. To our best knowledge, the results presented in this paper (patent submission PCT-EP2004-0012134) are the first to indicate that HDAC inhibition is, next to acquiring and maintaining differentiated functions in primary cells and cell lines, a key-factor to differentiate bone marrow-derived hMSC into functional hepatocyte-like cells, particularly under sequential culture conditions mimicking *in vivo *hepatogenesis.

Long-term cultivation and successful transdifferentiation of hMSC could be shown under both set-ups (sequential TSA and cocktail TSA), though a relatively high number (about 20%) of TSA-treated cells died, due to apoptosis. A feasible explanation could be that TSA, accompanied by hepatogenic agents, promotes selective survival of differentiating cells towards the hepatocyte lineage by inducing apoptosis of non- (hepatic) differentiating cells.

The established model opens new perspectives: it offers an unlimited source of functional human hepatocyte-like cells of non-embryonic origin, applicable in basic research (developmental endoderm biology), preclinical drug discovery and development, and cell and organ design. It might open a road for 'trans'-differentiation of cell types from different tissues towards endodermal lineages. In addition, as a consequence of their extensive self-renewing capacity, these cells may, in future, overcome the shortage of human donor tissue contributing as such to tissue regeneration in congenital and degenerative disorders.

## Methods

### Expansion of hMSC

Bone marrow samples were aspirated from the sternum of healthy (male and female) donors with variable age (35–85 years old), following informed consent. Bone marrow mononucleated cells were isolated by Ficoll-Hypaque (Nycomed, Lucron Bioproducts, De Pinte, Belgium) density-gradient centrifugation and plated in Mesenchymal Stem Cell Growth Medium (MSCGM, Cambrex, Verviers, Belgium) at 37°C and under 5%CO_2 _and 95%air After 4 hours, medium was renewed to remove non-adherent cells. At 50% confluency, cells were detached with trypsin (0.05% w/v)/EDTA (0.02% w/v) (Cambrex). Cells were cultured for 4 passages and finally harvested for further experiments.

### Characterization of culture-expanded hMSC

#### Immunophenotypic analysis

Cells were labeled with CD34-FITC [Becton Dickinson (BD), Erembodegem, Belgium], CD45-PE (DAKO), CD105-FITC (Ancell-10P's, Zandhoven, Belgium), CD73-PE, CD90-PE, CD166-PE (all from PharMingen, BD) and analyzed using a flow cytometer (Coulter Epics^® ^XL-MCL, Analis, Namur, Belgium).

#### Routine analysis of mesodermal potency

Cells were cultivated in specific media for adipogenic, osteogenic and chrondrogenic differentiation according to the manufacturer's instructions (Cambrex). Mesodermal differentiation was verified by means of different stainings (described below). To exclude spontaneous differentiation, hMSC were cultivated in regular MSCGM medium (Cambrex) and analyzed in parallel.

### Endodermal (hepatocyte) differentiation of hMSC

hMSC were plated at 21.5 10^3 ^cells/cm^2 ^on 1 mg/ml collagen gel type I (BD) in basal medium supplemented with 2% (v/v) foetal bovine serum (Hyclone, Perbio Science, Erembodegem, Belgium). Basal medium consisted of 60% (v/v) DMEM and 40% (v/v) MCDB-201 (Invitrogen, Merelbeke, Belgium) supplemented with 100 IE/ml penicillin (Continental Pharma, Diegem, Belgium), 100 μg/ml streptomycin, 1 mg/ml linoleic-acid bovine serum albumin, 0.1 mM L-ascorbic acid, 0.03 mM nicotinamide, 0.25 mM sodium pyruvate and 1.623 mM glutamine (all from Sigma, Bornem, Belgium). At 100% confluence, cells were exposed to hepatogenic cytokines and growth factors, added either simultaneous as a cocktail ('referred to as 'cocktail-condition/-set-up/-exposure' or a derivative thereof) [basal medium + 10 ng/ml FGF-4, 20 ng/ml HGF (all from R&D Systems, Minneapolis, USA), 1 × ITS and 20 μg/l dexamethasone (all from Sigma)] or sequentially ('referred to as 'sequential-condition/-set-up/-exposure' or a derivative thereof) (days 0–2: basal medium + 10 ng/ml FGF-4; days 3–5: basal medium + 20 ng/ml HGF; from day 6 on: basal medium + 20 ng/ml HGF + 1 × ITS and 20 μg/l dexamethasone). Differentiation media were changed every 3 days. From day 6 onwards, 1 μM TSA (Sigma) was added. The cocktail- and sequential-conditions, supplemented with 1 μM TSA will be further referred to as 'cocktail TSA-condition/-set-up/-exposure', and 'sequential TSA condition/-set-up/-exposure, respectively, or a derivative hereof.

### Staining

#### Cytological staining

Cells were fixed with 10% (w/v) formalin (PolySciences, Omnilabo International N.V. Aartselaar, Belgium) for 10 min at room temperature (adipogenic, hepatocyte differentiation) or with methanol (Merck, Overijse, Belgium) for 2 min at -20°C (osteogenic differentiation). After fixation, adipocytes were identified as red-colored lipid droplets upon staining with Oil-red O (from Sigma) [24]. Mineralized nodules or calcium deposits in differentiated osteogenic cells were stained black with the von Kossa technique [24]. Periodic-acid-Schiff (PAS) staining was used to determine glycogen storage, a functional parameter of endogenic differentiation [12]. As a control, PAS staining was performed in the presence of amyloglucosidase (Sigma).

#### Immunohistochemical staining

Chondrocytic differentiation was determined using rabbit polyclonal anti-collagen II antibody (NCL-COLL-IIp, NovoCastra, Prosan, Merelbeke, Belgium).

#### Immunofluorescence

Cells were fixed either with ethanol (Merck) for 10 min at -20°C (cytoskeletal proteins) or with 4% (w/v) paraformaldehyde (Electron Microscopy Sciences, Fort Washington, PA) for 10 min at 4°C (nuclear and cytoplasmic markers). After fixation, liver-specific protein expression was analysed using primary antibodies against AFP (goat), (HNF3β (goat), HNF1α (rabbit), C/EBPα (rabbit), MRP2 (goat; all from Santa Cruz, Heidelberg, Germany), CK18 (mouse, FITC-conjugated; Sigma) and ALB (goat, FITC-conjugated; Bethyl Laboratories Montgomery, TX). HDAC inhibition was assessed using anti-acetyl histone H4 antibody (rabbit, Upstate Biotechnology, NY, USA). Respective secondary antibodies came from Jackson Immunoresearch, Cambridgeshire, UK. As a negative control, cells were incubated with appropriate gamma immunoglobulines (Jackson Immunoresearch) and immunostained under the same conditions. Cells were analysed using fluorescence microscopy with a Zeiss Axiovert scope.

### Microscopic analysis

Cell morphology was analysed using phase-contrast light-microscopy (Nikon).

### Albumin ELISA

ALB concentrations, secreted into the culture media, were analysed by ELISA [44].

### Urea assay

Urea concentrations produced, after 24 hours-exposure of the cells to 6 mM NH_4_Cl (Sigma), were measured colometrically in the culture media according to the manufacturer's instructions (Quantichrom Urea assay kit, Bioassay Systems, Brussels, Belgium). Fresh culture media supplemented with 6 mM NH_4_Cl (Sigma) and 4 hours-cultured adult hepatocytes were used as negative and positive controls, respectively.

### Alkoxyresorufin-O-dealkylase assay

EROD- and PROD-activities were assessed as previously described [45] with some minor modifications: in our set-up, cells were incubated with 20 μM 7-ethoxyresorufin and 18 μM 7-pentoxyresorufin (all from Sigma) for 3 hours and subsequently for 30 min with β-glucuronidase/arylsulfatase (Roche Applied Science, Vilvoorde, Belgium).

To evaluate the inducibility of CYP2B6 and CYP1A1/2, cells were, after 18 days of differentiation, exposed to PB (final concentration 1 mM) and MC (final concentration 2 μM; all from Sigma), respectively. Media, supplemented with either PB or MC, were daily renewed. Fresh culture media and 4 hours-cultured adult hepatocytes were used as negative and positive controls, respectively.

### Statistics

Results are expressed as mean ± SD. Statistical analyses were performed using Oneway Anova and Student's t-test. The significance level was set at 0.05.

## Abbreviations

Albumin (ALB); alpha-fetoprotein (AFP); CCAAT-enhancer binding protein (C/EBP); cytochrome P450 (CYP); cytokeratin (CK); dimethylsulfoxide (DMSO); ethoxyresorufin-O-deethylase (EROD); fibroblast growth factor-4 (FGF-4); hepatocyte growth factor (HGF); hepatocyte nuclear factor (HNF); histone deacetylase inhibitor (HDAC-I); human adipose tissue-derived stromal cells (hADSC); human mesenchymal stem cells (hMSC); insulin-transferrin-sodium-selenite (ITS); lactate dehydrogenase (LDH); multidrug resistance-associated protein (MRP); multipotent adult progenitor cells (MAPC); methylcholantrene (MC); new chemical entities (NCEs); oncostatin M (OSM); pentoxyresorufin-O-dealkylase (PROD); Periodic-acid-Schiff (PAS) staining; phenobarbital (PB); trichostatin A (TSA).

## Authors' contributions

**SS**: Differentiation of expanded hMSC under hepatocyte-specific conditions. Analysis of multilineage (adipogenic, osteogenic, neuroectodermal and endodermal) differentiation by means of staining, functionality assays, microscopic analysis. Preparation of the manuscript.

**TV, PP and MV**: Optimization of the concentration of TSA and onset of exposure to TSA. Critical revision of the manuscript. **ADB and IVR**: Isolation, expansion and routine characterization of hMSC (staining, immunophenoptypic analysis). Critical revision of the manuscript. **VR**: Head of department of Toxicology and promotor of the hereby associated PhD thesis. Microscopic analysis. Critical revision of the manuscript.

All authors read and approved the final manuscript.

## Supplementary Material

Additional file 1 DNA synthesis of sequentially (+/-1 μM TSA) and cocktail (+/-1 μM TSA)-exposed hMSC. hMSC, plated on 1 mg/ml collagen gel type I, were at 100% confluence treated with either the cocktail- or sequential-condition. From day 6 onwards, 1 μM TSA was added ('cocktail TSA' and 'sequential TSA' conditions). Differentiation media were changed every 3 days. Samples for DNA synthesis were taken 2, 4, 6, 12 and 18 hours upon media change. [Methyl-^3^H] thymidine (25 μCi/mmol, 2 μCi/ml) incorporation into trichloroacetic acid precipitated-DNA (Amersham Inc, Diegem, Belgium) was measured using a scintillation counter (Wallac 1410). Graphs shown are representative for 3 independent experiments. Click here for file

Additional file 2 Cell viability analysis of sequentially (+/-1 μM TSA) and cocktail (+/-1 μM TSA)-exposed hMSC. hMSC, plated on 1 mg/ml collagen gel type I, were at 100% confluence treated with either the cocktail- or sequential-condition. From day 6 onwards, 1 μM TSA was added ('cocktail TSA' and 'sequential TSA' conditions). LDH leakage into culture media was measured throughout culture time according to the Bergmeyer procedure (Bergmeyer, 1974) using a commercial kit (Merck, Germany). Graphs shown are representative for at least 3 independent experiments. Click here for file

Additional file 3 Cell death analysis of sequentially (+/-1 μM TSA) and cocktail (+/-1 μM TSA)-exposed hMSC. hMSC, plated on 1 mg/ml collagen gel type I, were at 100% confluence treated with either the cocktail- or sequential-condition. From day 6 onwards, 1 μM TSA was added ('cocktail TSA' and 'sequential TSA' conditions). Differentiation media were changed every 3 days. Cells were, 12 hours upon media change, incubated with Alexa Fluor 488 annexin V (green fluorescent), propidiumiodide (red fluorescent) and the nuclear counterstain DAPI (blue fluorescent). The red, green, and both red and green-stained cells represent necrotic, apoptotic and death cells, respectively. 20 × 10 original magnification, phase contrast. Stainings shown are representative for at least 3 separate experiments. Click here for file

## References

[B1] Ragan I (2006). The innovative medicines initiative: a proposal with implications for the 3R's. NC3Rs.

[B2] Gomez-Lechon MJ, Donato MT, Castell JV, Jover R (2003). Human hepatocytes as a tool for studying toxicity and drug metabolism. Curr Drug Metab.

[B3] Gomez-Lechon MJ, Donato MT, Ponsoda X, Castell JV (2003). Human hepatic cell cultures: in vitro and in vivo drug metabolism. Altern Lab Anim.

[B4] Papeleu P, Vanhaecke T, Rogiers V (2006). Histone deacetylase inhibition: A differentiation therapy for cultured primary hepatocytes?. Current Enzyme Inhibition.

[B5] Papeleu P, Loyer P, Vanhaecke T, Elaut G, Geerts A, Guguen-Guillouzo C, Rogiers V (2003). Trichostatin A induces differential cell cycle arrests but does not induce apoptosis in primary cultures of mitogen-stimulated rat hepatocytes. J Hepatol.

[B6] Rogiers V, Vercruysse A (1998). Hepatocyte cultures in drug metabolism and toxicological research and testing. Methods Mol Biol.

[B7] LeCluysse EL, Bullock PL, Parkinson A (1995). Strategies for restoration and maintenance of normal hepatic structure and function in long-term cultures of rat hepatocytes. Adv Drug Del Rev.

[B8] Krause DS, Theise ND, Collector MI, Henegariu O, Hwang S, Gardner R, Neutzel S, Sharkis SJ (2001). Multi-organ, multi-lineage engraftment by a single bone marrow-derived stem cell. Cell.

[B9] Huttmann A, Li CL, Duhrsen U (2003). Bone marrow-derived stem cells and plasticity. Ann Hematol.

[B10] Friedenstein AJ, Chailakhyan RK, Gerasimov UV (1987). Bone marrow osteogenic stem cells: in vitro cultivation and transplantation in diffusion chambers. Cell Tissue Kinet.

[B11] Friedenstein AJ, Gorskaja JF, Kulagina NN (1976). Fibroblast precursors in normal and irradiated mouse hematopoietic organs. Ex Hematol.

[B12] Schwartz RE, Reyes M, Koodie L, Jiang Y, Blackstadt M, Lund T, Lenvik T, Johnson S, Hu WS, Verfaillie CM (2002). Multipotent adult progenitor cells from bone marrow differentiate into functional hepatocyte-like cells. J Clin Invest.

[B13] Papeleu P, Elaut G, Rogiers V, Vanhaecke T, Pandalai SG (2002). Cell cultures as *in vitro *tools for biotransformation studies. Recent Research Developments in Drug Metabolism and Disposition.

[B14] Papeleu P, Vanhaecke T, Elaut G, Vinken M, Henkens T, Snykers S, Rogiers V (2005). Differential effects of histone deacetylase inhibitors in tumor and normal cells-what is the toxicological relevance?. Crit Rev Toxicol.

[B15] Vanhaecke T, Henkens T, Kass GE, Rogiers V (2004). Effect of the histone deacetylase inhibitor trichostatin A on spontaneous apoptosis in various types of adult rat hepatocyte cultures. Biochem Pharmacol.

[B16] Vanhaecke T, Papeleu P, Elaut G, Rogiers V (2004). Trichostatin A-like hydroxamate histone deacetylase inhibitors as therapeutic agents: toxicological point of view. Curr Med Chem.

[B17] Herold C, Ganslmayer M, Ocker M, Hermann M, Geerts A, Hahn EG, Schuppan D (2002). The histone-deacetylase inhibitor Trichostatin A blocks proliferation and triggers apoptotic programs in hepatoma cells. J Hepatol.

[B18] Yamashita Y, Shimada Y, Harimoto N, Rikimura T, Shirabe T, Tanaka S, Sugimachi K (2003). Histone deacetylase inhibitor trichostatin A induces cell-cycle arrest/apoptosis and hepatocyte differentiation in human hepatoma cells. Int J Cancer.

[B19] Chiba T, Yokosuka O, Fukai K, Kojima H, Tada M, Arai M, Imazeki F, Saisho H (2004). Cell growth inhibition and gene expression induced by the histone deacetylase inhibitor, trichostatin A, on human hepatoma cells. Oncology.

[B20] Snykers S, Vanhaecke T, Papeleu P, Luttun A, Jiang Y, Vander Heyden Y, Verfaillie C, Rogiers V (2006). Sequential Exposure to Cytokines Reflecting Embryogenesis: The Key for in vitro Differentiation of Adult Bone Marrow Stem Cells into Functional Hepatocyte-like Cells. Toxicol Sci.

[B21] Shen S, Li J, Casaccia-Bonnefil P (2005). Histone modifications affect timing of oligodendrocyte progenitor differentiation in the developing rat brain. J Cell Biol.

[B22] Gomez-Lechon MJ, Donato MT, Castell JV, Jover R (2004). Human hepatocytes in primary culture: the choice to investigate drug metabolism in man. Curr Drug Metab.

[B23] Rogiers V, Vercruysse A (1993). Rat hepatocyte cultures and co-cultures in biotransformation studies of xenobiotics. Toxicology.

[B24] Reyes M, Lund T, Lenvik T, Aguiar D, Koodie L, Verfaillie CM (2001). Purification and ex vivo expansion of postnatal human marrow mesodermal progenitor cells. Blood.

[B25] Jiang Y, Henderson D, Blackstad M, Chen A, Miller RF, Verfaillie CM (2003). Neuroectodermal differentiation from mouse multipotent adult progenitor cells. PNAS.

[B26] Henningson CT, Stanislaus MA, Gewirtz AM (2003). Embryonic and adult stem cell therapy. J Allergy Clin Immunol.

[B27] Henkens T, Papeleu P, Elaut G, Vinken M, Rogiers V, Vanhaecke T Trichostatin A, a critical factor in maintaining the functional differentiation of primary cultured rat hepatocytes. Toxicol Appl Pharmacol.

[B28] Vinken M, Henkens T, Vanhaecke T, Papeleu P, Geerts A, Van Rossen E, Chipman JK, Meda P, Rogiers V (2006). Trichostatin A enhances gap junctional intercellular communication in primary cultures of adult rat hepatocytes. Toxicol Sci.

[B29] Cascio S, Zaret KS (1991). Hepatocyte differentiation initiates during endodermal-mesenchymal interactions prior to liver formation. Development.

[B30] Shiojiri N (1981). Enzymo- and immunocytochemical analyses of the differentiation of liver cells in the prenatal mouse. J Embryol Exp Morphol.

[B31] Ott MO, Rey-Campos J, Cereghini S, Yaniv M (1991). vHNF1 is expressed in epithelial cells of distinct embryonic origin during development and precedes HNF1 expression. Mech Dev.

[B32] Gao B, St Pierre MV, Stieger B, Meier PJ (2004). Differential expression of bile salt and organic anion transporters in developing rat liver. J Hepatol.

[B33] Seo MJ, Suh SY, Bae YC, Jung JS (2005). Differentiation of human adipose stromal cells into hepatic lineage in vitro and in vivo. Biochem Biophys Res Commun.

[B34] Lee KD, Kuo TK, Whang-Peng J, Chung YF, Lin CT, Chou SH, Chen JR, Chen YP, Lee OK (2004). In vitro hepatic differentiation of human mesenchymal stem cells. Hepatology.

[B35] Kang XQ, Zang WJ, Song TS, Xu XL, Yu XJ, Li DL, Meng KW, Wu SL, Zhao ZY (2005). Rat bone marrow mesenchymal stem cells differentiate into hepatocytes in vitro. World J Gastroenterol.

[B36] Kang XQ, Zang WJ, Bao LJ, Li DL, Song TS, Xu XL, Yu XJ (2005). Fibroblast growth factor-4 and hepatocyte growth factor induce differentiation of human umbilical cord blood-derived mesenchymal stem cells into hepatocytes. World J Gastroenterol.

[B37] Hong SH, Gang EJ, Jeong JA, Ahn C, Hwang SH, Yang IH, Park HK, Han H, Kim H (2005). In vitro differentiation of human umbilical cord blood-derived mesenchymal stem cells into hepatocyte-like cells. Biochem Biophys Res Commun.

[B38] Lange C, Bassler P, Lioznov MV, Bruns H, Kluth D, Zander AR, Fiegel HC (2005). Liver-specific gene expression in mesenchymal stem cells is induced by liver cells. World J Gastroenterol.

[B39] Aurich I, Mueller LP, Aurich H, Luetzkendorf J, Tisljar K, Dollinger M, Schormann W, Walldorf J, Hengstler J, Fleig WE, Christ B (2006). Functional integration of human mesenchymal stem cell-derived hepatocytes into mouse livers. Gut.

[B40] Li W, Liu SN, Luo DD, Zhao L, Zeng LL, Zhang SL, Li S (2006). Differentiation of hepatocytoid cell induced from whole-bone-marrow method isolated rat myeloid mesenchymal stem cells. World J Gastroenterol.

[B41] Ong SY, Dai H, Leong KW (2006). Inducing hepatic differentiation of human mesenchymal stem cells in pellet culture. Biomaterials.

[B42] Sarg B, Helliger W, Talasz H, Koutzamani E, Lindner HH (2004). Histone H4 hyperacetylation precludes histone H4 lysine 20 trimethylation. J Biol Chem.

[B43] D'Ippolito G, Schiller PC, Ricordi C, Roos BA, Howard GA (1999). Age-related osteogenic potential of mesenchymal stromal stem cells from human vertebral bone marrow. J Bone Miner Res.

[B44] Koebe HG, Wick M, Cramer U, Lange V, Schildberg FW (1994). Collagen gel immobilisation provides a suitable cell matrix for long term human hepatocyte cultures in hybrid reactors. Int J Artif Organs.

[B45] Donato MT, Gomez-Lechon MJ, Castell JV (1993). A microassay for measuring cytochrome P450IA1 and P450IIB1 activities in intact human and rat hepatocytes cultured on 96-well plates. Anal Biochem.

