# Supplementary figures and images for: Chromatin remodeling agent trichostatin A: a key-factor in the hepatic differentiation of human mesenchymal stem cells derived of adult bone marrow
Source: BMC Dev Biol. 2007 Apr 2;7:24. doi: 10.1186/1471-213X-7-24 (PMC1852547; doi:10.1186/1471-213X-7-24)

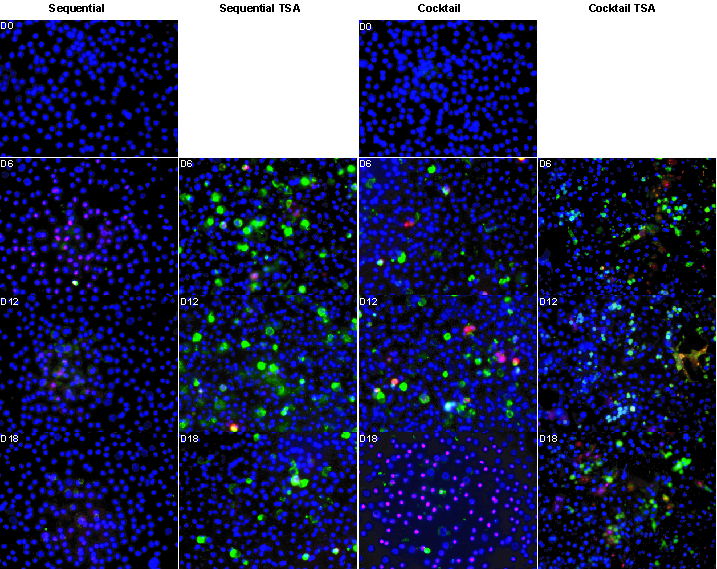

Supplement: Additional file 3 — Cell death analysis of sequentially (+/-1 μM TSA) and cocktail (+/-1 μM TSA)-exposed hMSC. hMSC, plated on 1 mg/ml collagen gel type I, were at 100% confluence treated with either the cocktail- or sequential-condition. From day 6 onwards, 1 μM TSA was added ('cocktail TSA' and 'sequential TSA' conditions). Differentiation media were changed every 3 days. Cells were, 12 hours upon media change, incubated with Alexa Fluor 488 annexin V (green fluorescent), propidiumiodide (red fluorescent) and the nuclear counterstain DAPI (blue fluorescent). The red, green, and both red and green-stained cells represent necrotic, apoptotic and death cells, respectively. 20 × 10 original magnification, phase contrast. Stainings shown are representative for at least 3 separate experiments. [file 1471-213X-7-24-S3.jpeg]
